# First Indonesian report of WGS-based MTBC L3 discovery

**DOI:** 10.1186/s13104-024-06825-5

**Published:** 2024-06-25

**Authors:** Yuni Rukminiati, Felix Mesak, Dina Lolong, Pratiwi Sudarmono

**Affiliations:** 1 Doctoral Program in Biomedical Sciences, Faculty of Medicine, University of Indonesia, Jakarta, Indonesia; 2 National Laboratory of Prof Sri Oemijati, Ministry of Health of the Republic of Indonesia, Jakarta, Indonesia; 3 National Research and Innovation Agency, Jakarta, Indonesia; 4 Department of Clinical Microbiology, Faculty of Medicine, University of Indonesia, Jakarta, Indonesia

**Keywords:** Tuberculosis, Whole genome sequencing, Phylogenomic

## Abstract

**Objective:**

Recent spoligotyping results in the island nation of Indonesia had revealed the existence of *Mycobacterium tuberculosis* complex lineage 3 (MTBC L3) or Central Asian (CAS) strains. In this work, whole-genome sequencing (WGS) – based methods were used to search for the presence of MTBC L3.

**Results:**

Two unrelated Indonesian L3 strains discovered by WGS-based SNP phylogenomics are presented here for the first time. Assemblies of their genomes yielded 96.95% (MTBC strain Mtb*_*S6970) and 98.35% (Mtb*_*S19106) of the known reference strain H37Rv. Their respective constructed genome coverages are 45.38 *±* 12.95x and 63.13 *±* 21.10x. The two L3 genomes have 4062 and 4121 genes, respectively, which are well within the number of genes predicted in MTBC strains. Instead of having three rRNA genes usually, Mtb_S6970 possesses four. These L3 isolates exhibit cross-class antibiotic susceptibility. *FadD26*, *fadE24*, *fbpA*, *lprO*, and *panC*, which are thought to be important in the pathophysiology of MTBC, were discovered to have 3–7 times more loci in L3 than L2 or L4. The penetration of L3 in the nation, despite its antibiotic sensitivity, is a concerning indicator of borderless global spread that may eventually be overcome by the phenotypes of acquired drug resistance.

**Supplementary Information:**

The online version contains supplementary material available at 10.1186/s13104-024-06825-5.

## Introduction

**Fig. 1:**
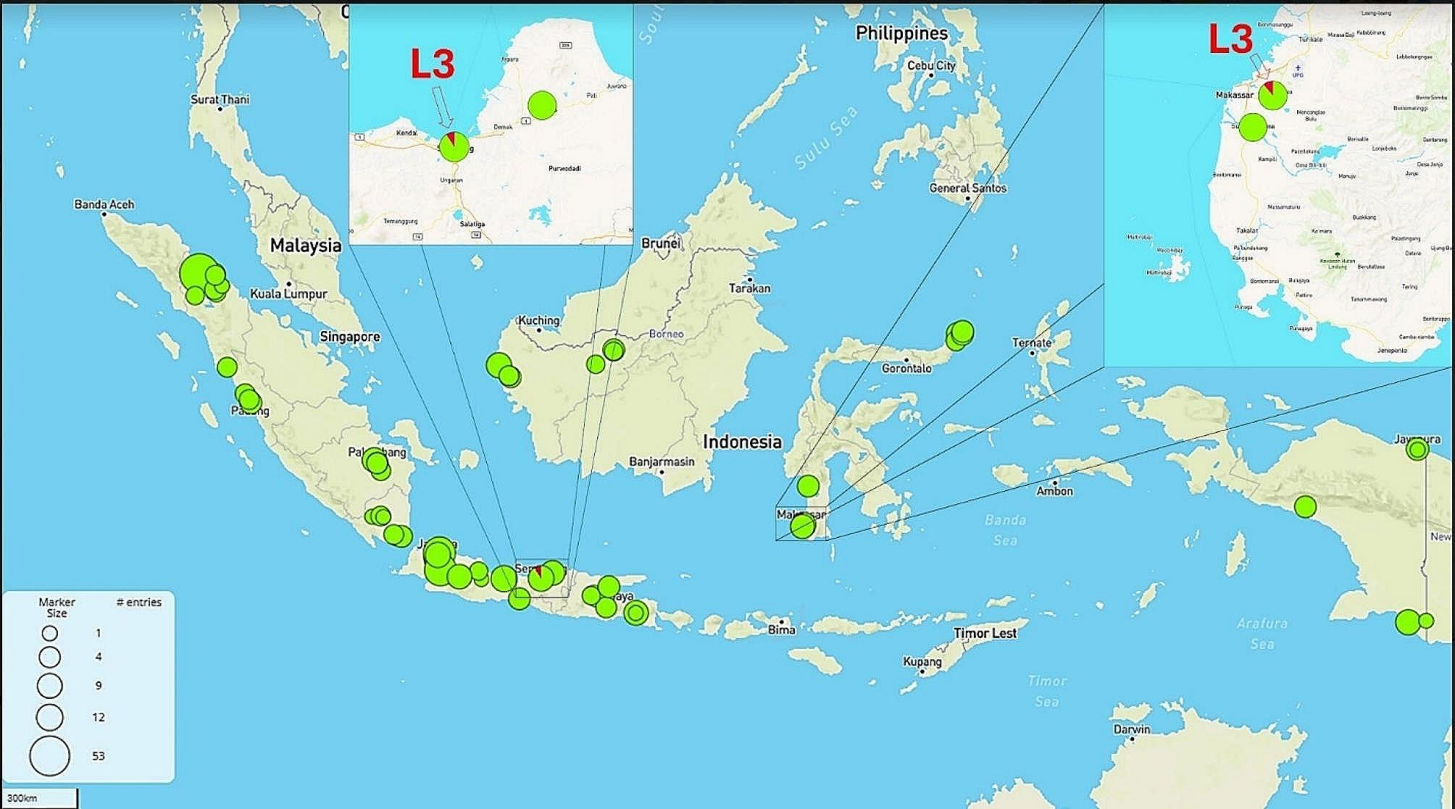
A map of the Indonesian archipelago showing the locations of the samples taken for the WGS of MTBC.

*Mycobacterium tuberculosis sensu stricto* and *M. africanum*, these strains are what most cause tuberculosis in humans [1]. *Mycobacterium tuberculosis* complex (MTBC) species are classified into seven lineages, with the eighth (as well as the ninth as a descendant of the sixth) being proposed lately [2–4]. Although a few human-adapted MTBC lineages are still geographically restricted, the vast majority of lineages have now or may soon spread globally, including Lineage 1 (L1) or the Indo-Oceanic and East African-Indian (EAI) strains, L2 or the East Asian Beijing strains, L3 or Central Asian (CAS) strains, L4 or Euro-American Haarlem, LAM, X, T, C strains, L5 and L6 or West African 1 and 2 *M. africanum* strains, L7 or the Ethiopian strains, and L8 or the Rwandan and Ugandan strains [4]. This study will continue to refer to the lineages by their numerical designation, i.e., L1–8 according to Fig. 1 at [4], in order to prevent naming confusion for the MTBC lineages [4].

Ruesen et al. (2018) used whole genome sequencing (WGS) analysis to identify the MTBC L1, L2, and L4 at 2.5%, 36.0%, and 61.5%, respectively, among 322 isolates [5]. Maladan et al. (2021) additionally identified MTBC L1, L2 and L4 at 36.8%, 26.3%, and 36.8%, respectively, among 19 isolates acquired exclusively from an Indonesian island of Papua [6]. This was in keeping with the widespread deployment of WGS analysis to identify MTBC lineages in Indonesia. Unfortunately, neither of the two WGS investigations found any L3 [5, 6]. This happened because the number of samples was small so that L3 was not found in the study. Although the percentages of MTBC lineages discovered in prior studies vary, it is clear that the most common MTBC lineages detected thus far are L1, L2, and L4. In order to accurately map the distribution of MTBC lineages in the archipelagic nation, a statistically larger sample size is fundamentally required. MTBC isolates gathered from several islands across the nation are currently being subjected to whole genome sequencing study by the Ministry of Health. Two L3 strains, which are being reported here for the first time, were found among the 1128 sequenced isolates.

## Materials and methods

### Species determination and antibiotic susceptibility assay

The MTBC species and resistance to the first line of antibiotics, rifampicin, were determined by a DNA-based assay using Xpert® MTB/RIF (Cepheid, Sunnyvale, CA, USA). The laboratory protocol was carried out in accordance with the manufacturer’s recommendations. Sputum samples containing positive MTBC were then cultured and sensitivity assay for first line drugs (Streptomycin, isoniazid, rifampicin and ethambutol) and second line drugs (Ofloxacin, amikacin and kanamycin) used BD BACTEC™ MGIT™ 960 system (Becton, Dickinson and Company, Franklin Lakes, NJ, USA). All cultivated samples were kept in a deep freezer at -80^o^C.

### Re-culturing MTBC bacteria

Löwenstein-Jensen (LJ) agar medium was used to cultivate 0.1mL patient samples that were taken from the repository of Indonesian Tuberculosis’ Drug Resistance Survey. After 2 to 4 weeks of incubation, colonies were scraped and transferred to a microcentrifuge tube with 1x TE Buffer solution. The suspension was heated to 95 °C for 30 min to kill the bacteria. For total DNA isolation and whole genome sequencing, the suspension samples were transported to the bacteriology lab of the Research and Development Center for Basic Health Technology.

### Isolation and purification of total MTBC DNA

Total DNA isolation of MTBC bacteria were accomplished using the N-cetyl-N, N,N-trimethyl ammonium bromide (CTAB) protocol [7]. Afterward, Qubit Fluorometer 4.0 and Nanodrop were used to measure the quantity and quality of the isolated DNA, respectively. The limit of DNA quantity and purity is set to *≥* 0.2 ng/μl and 1.7 *≥* A_260_/A_280_ *≤* 2.0, respectively.

### WGS library preparation

The Nextera XT DNA Library Preparation Kit 2 × 300 bp and Miseq Reagent Kit V3 (600 cycle) from Illumina, San Diego, CA, USA, were used with the manufacturer’s instructions.

### MTBC reference genomes

Raw sequence reads as MTBC references for wildtype and lineage genomes were obtained from the Sequence Read Archive (SRA, https://www.ncbi.nlm.nih.gov/sra). A full genome of wildtype *Mycobacterium tuberculosis* H37Rv with the GenBank accession number NC 000962 was utilized as the reference genome for this work. The raw sequencing data for the reference genome’s paired sequencing read was downloaded from the SRA with the run number. (Table 1)

**Table 1:** Raw sequence reads as MTBC references for wildtype and lineage genomes

SRA	Lineage	deposited by	Design as
SRR23315112		Amsterdam University Medical Center in the Netherlands	MtbH37Rv_NED1_SRR23315112
ERR3273086	L4	Wellcome Sanger Institute, UK	Mtb_IDN1_ERR3273086
SRR12416847	L2	Khon Kaen University in Thailand	Mtb_IDN2_SRR12416847
SRR18548950	L2	Chongqing Medical University’s Children’s Hospital, China	Mtb_CHN1_SRR18548950
SRR22424726	L2	National Tuberculosis Institute, Bangalore, India	Mtb_IND1_SRR22424726
SRR23174261		Agrifood and Biosciences Institute, Ireland	Mbovis_IRE1_SRR23174261
ERR266106		Institut de Génétique et Microbiologie, Université Paris Sud, Orsay, France	Mcanettii_DJI2_ERR266106
ERR2245380	L3	Norwegian Institute of Public Health	Mtb_NOR1_ERR2245380
ERR038743, ERR040140, ERR2179842	L3	Uganda	
ERR9030282, ERR9030307, ERR9030352, ERR9030357, ERR9030453, ERR9030473, ERR9030486, ERR9030525;	L3	Madagascar	
SRR5341388	L3	India	
SRR10147700	L3	Iran	
SRR6046358, SRR6046512, SRR7069090, SRR7069750, SRR7069770, SRR7070466	L3	UK	
SRR6152977, SRR6153073, SRR6397682	L3	Canada	
ERR2245422, ERR2245424, ERR2245426, ERR2512375, ERR2512377	L3	Norway	
ERR5979465, ERR5979467, ERR5979469, ERR5979471, ERR5979473.	L3	Germany	

### Bioinformatic analyses

Utilizing the public servers at usegalaxy.eu, the sequencing data were analyzed [8]. (More complete methods in supplementary)

## Results

### Indonesian L3 patients

Throughout this study, we found two MTBC L3 strains, *M. tuberculosis* S6970 and *M. tuberculosis* S19106 among sequenced isolates (Fig. 1). One was found in a patient in Central Java of Java Island (Mtb_S6970), and the other was from South Sulawesi of Sulawesi (Celebes) Island (Mtb_S19106), each being discovered 1400 km apart (Fig. 1). It is interesting to note that both strains were detected in males between the ages of 55 and 64, yet one was isolated from a non-smoker (Mtb_S19106), and the other was isolated from a heavy smoker (Mtb_S6970) (Table 2).

**Table 2:** An outline of the traits found in the genome comparison of the two Indonesian MTBC L3 strains, Mtb_S6970 and Mtb_S19406, and selected MTBC lineages

Attibutes	Tool	Mtb_S6970	Mtb_S19106	Mbovis_IRE1	Mtb-CHN1	Mtb_IDN1	Mtb-IDN2	Mtb_IND1	Mtb_NOR1	MtbH37Rv_NED1
SRA	n/a	SRR24682255	SRR24682254	ERR3273086	SRR18548950	ERR3273086	SRR12416847	SRR22424726	ERR2245380	SRR23315112
case Status	Survey	Retreated	New	n/a	n/a	n/a	n/a	n/a	n/a	n/a
Smoking status	Survey	Heavy smoker	Non smoker	n/a	n/a	n/a	n/a	n/a	n/a	n/a
Gender	Survey	Male	Male	n/a	n/a	n/a	n/a	n/a	n/a	n/a
Work status	Survey	Unemployed	Self-employed	n/a	n/a	n/a	n/a	n/a	n/a	n/a
Age group	Survey	55–64	55–64	n/a	n/a	n/a	n/a	n/a	n/a	n/a
Genome size (nt), reference	Unicycler, Quast	4,411,532	4,411,532	4,411,532	4,411,532	4,411,532	4,411,532	4,411,532	4,411,532	4,411,532
Genome size (nt)	Unicycler, Quast	4,277,172	4,338,867	4,228,622	4,350,790	4,348,793	4,352,042	4,264,381	4,376,429	4,238,850
Genome fraction (%)	Unicycler, Quast	96.95%	98.35%	95.85%	98.62%	98.58%	98.65%	96.66%	99.20%	96.09%
Genome coverage (x)	QualiMap	63.13 ± 21.10	45.38 ± 12.95	24.76 ± 7.81	150.45 ± 20.07	64.41 ± 12.48	186.37 ± 47.43	22.69 ± 8.24	36.46 ± 10.77	19.58 ± 7.10
#Contigs	Unicycler, Quast	152	167	103	144	106	129	197	151	192
Missassembled contigs length	Unicycler, Quast	1,434,759	1,160,296	1,976,600	807,473	1,519,622	1,111,146	983,066	1,302,254	157,029
Missassembled contigs (% reference)	Unicycler, Quast	32.52%	26.30%	44.81%	18.30%	34.45%	25.19%	22.28%	29.52%	3.56%
#CDS	Unicycler, Prokka	4,006	4,065	3,97	4,021	4,029	4,029	4,044	4,081	4,047
#Genes	Unicycler, Prokka	4,062	4,121	4,025	4,077	4,086	4,085	4,099	4,138	4,102
#rRNA	Unicycler, Prokka	4	3	3	3	3	3	3	4	3
#Repeat region	Unicycler, Prokka	3	3	2	1	2	1	2	1	2
#tRNA	Unicycler, Prokka	51	52	51	52	53	52	51	52	51
#tmRNA	Unicycler, Prokka	1	1	1	1	1	1	1	1	1
GC (%)	Unicycler, Prokka	65.50%	65.51%	65.43%	65.59%	65.60%	65.59%	65.40%	65.56%	65.37%
N50	Unicycler, Prokka	69,879	68,808	107,846	105,502	126,308	151,652	51,02	82,783	40,435
Lineage	TB Profiler	3	3	La.1.8.1	2	4	2	2	3	4
Drug resistance	TB Profiler	Sensitive	Sensitive	PZA	Sensitive	RIF	MDR: RIF, INH, EMB	Pre-XDR: FQs, RIF, STR, INH, EMB, PZA, KAN, PAS, ETA	Sensitive	LZD
Gene	TB Profiler	n/a	n/a	pncA	n/a	rpoB	rpoB; fabG1	gyrB; gyrA; rpoB; rpsL; fabG1; katG; pncA; eis; thyA; embA; ethA	n/a	rrl
Mutation	TB Profiler	n/a	n/a	p.His57Asp	n/a	p.His445Asn	p.Ser450Leu; c.-15 C > T	p.Arg446Cys; p.Asp94Ala; p.Ser450Leu; p.Lys43Arg; c.-8T > C; p.Ser315Thr; p.Ile5Ser; c.-10G > A; p.Thr22Ala; c.-16 C > T; p.Trp391*	n/a	n.2299G > T
Type	TB Profiler	n/a	n/a	missense variant	n/a	missense variant	missense variant; upstream gene variant	missense variant; upstream gene variant; stop gained	n/a	non coding transcript exon variant
Frequency (%)	TB Profiler	n/a	n/a	100%	n/a	98%	100%	100%	n/a	11%

### Genomes of Indonesian MTBC L3

**Fig. 2:**
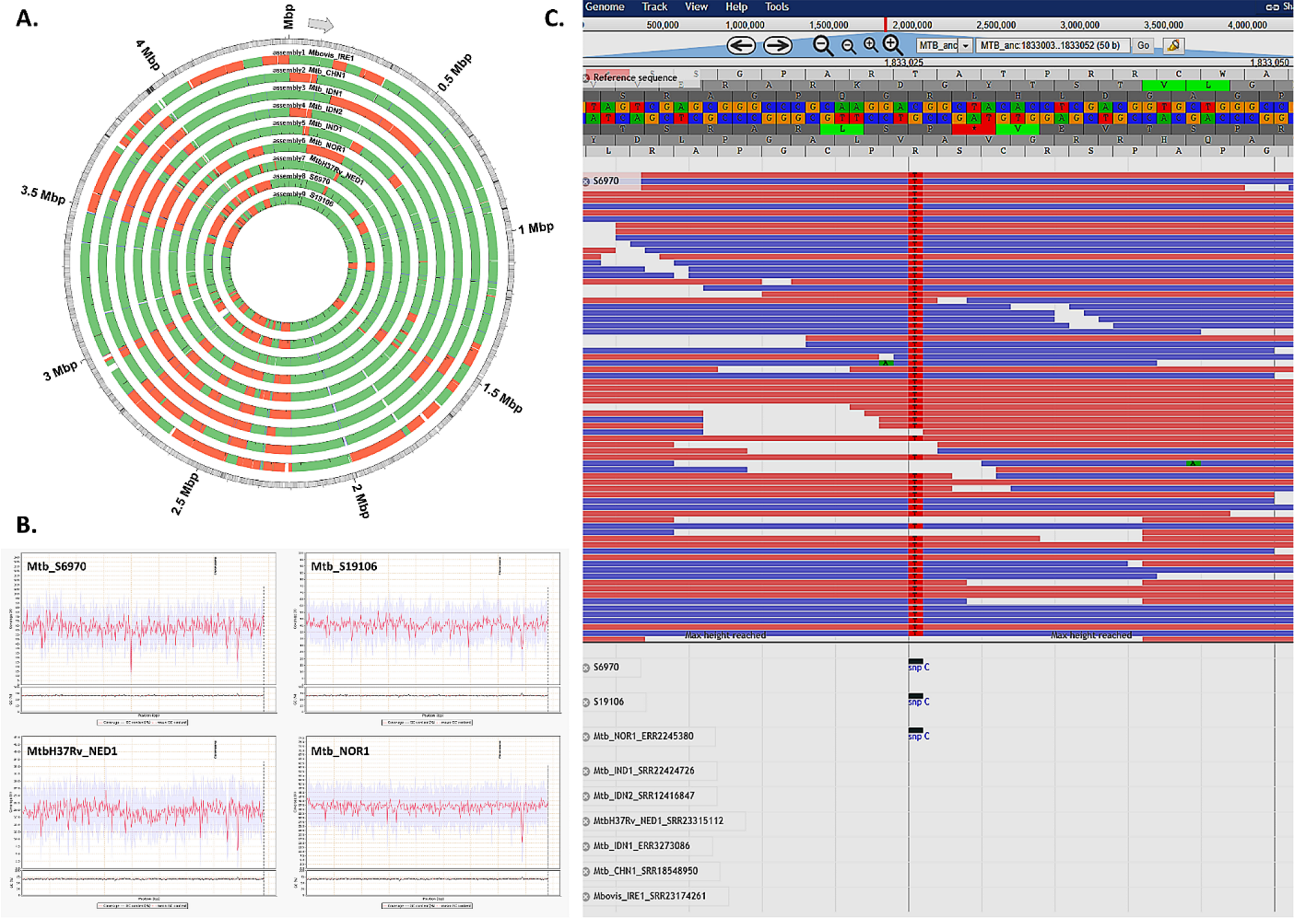
Comparative bioinformatics analysis of the MTBC L3 genomes from Indonesia to known L3 and other lineages. **(A)** An illustration of the alignment of several assembled genomes with respect to the known MTBC ancestral sequence H37Rv (represented by the outer ring shaded in grayscale). **(B)** Representative graphs of the MTBC L3 genome coverage produced from the qualimap pipeline’s assessment of the sequencing reads’ quality [21]. **(C)** The jbrowse package created a visualization image of unicycler assembled and prokka annotated genomic sequences [11, 22, 23]

The lineage family of the samples was identified using SNP phylogenomics analysis of the entire genome sequences of the L3 isolates and numerous well-known references for L2, L3, and L4, including the ancestor or reference genome of MTBC (For an illustration of a SNP present only in the L3, see Fig. 2C). While the controls H37Rv and known L3 Mtb_NOR1 had their average genome coverage at approximately 20x and 36x, respectively, the two Indonesian MTBC L3 had higher ones at 63x for Mtb_S6970 and 45x for Mtb_S19106. Better sequencing quality can be inferred from the assembled genome’s higher coverage of the reference sequence (Fig. 2B). It is feasible to exclude a specific polymorphism that is unique to MTBC L3 in comparison to the other lineages. As an illustration, a special single nucleotide polymorphism (SNP) found in all three MTBC L3 lineages (Mtb_S6970, Mtb_S19106, Mtb_NOR1) but not in the other lineages is presented. Undoubtedly, a focused examination of a very large number of isolates from diverse geographical locations is required to confirm the presence of such polymorphism in a given lineage.

### Indonesian MTBC L3 phylogeny

**Fig. 3:**
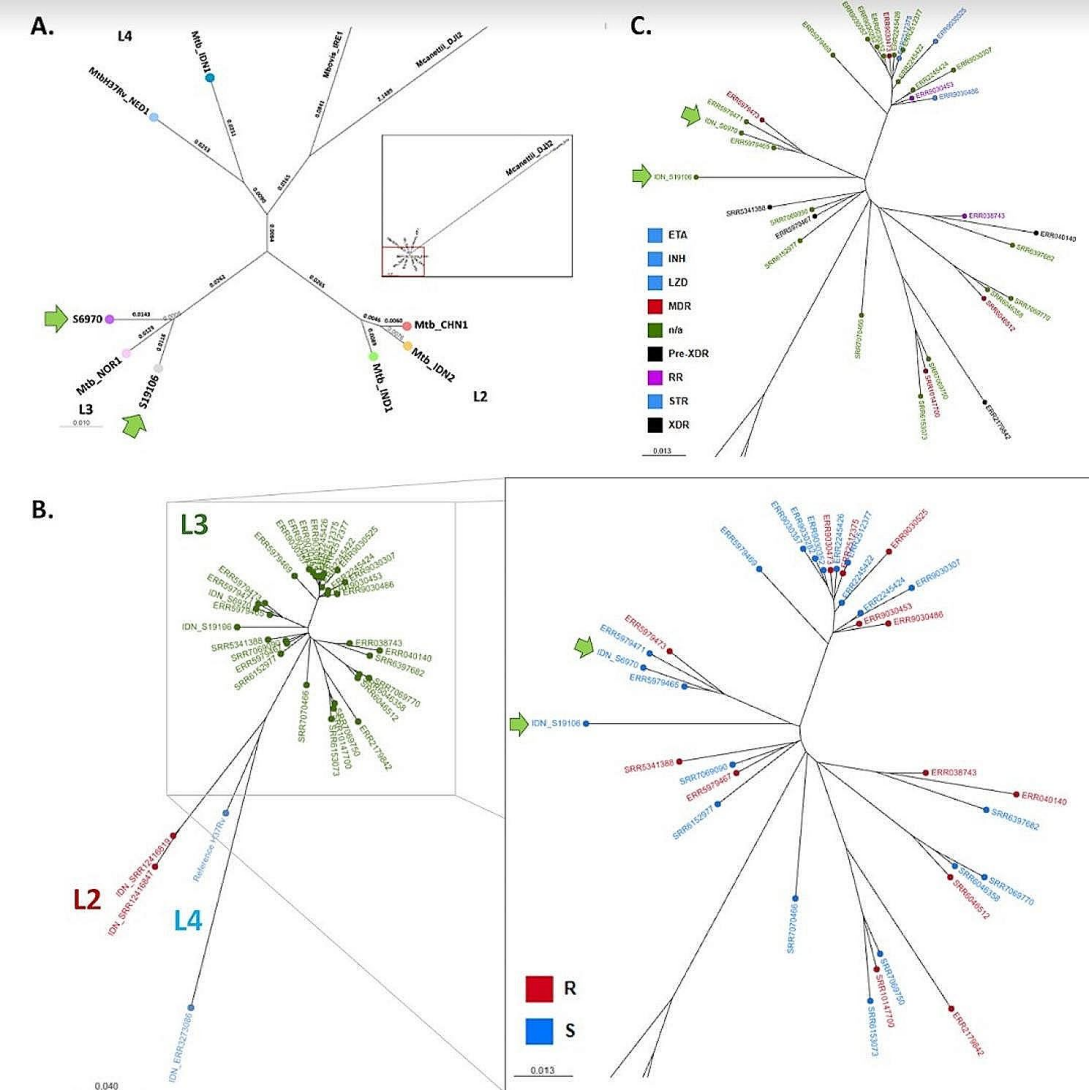
Phylogenetic tree images. **(A)** SNPs phylogenomic tree of tested MTBC L3 genomes against its L3, L2, and L4 known controls, unrelated *M. bovis* genome, and rooted in *M. canettii* genome. **(B)** To further validate the discovery, 32 MTBC L3 WGS raw reads from throughout the world were randomly selected and submitted to a phylogenomics study, including the two Indonesian MTBC L3 strains as well as known L2 and L4 strains. **(C)** The different levels of antibiotic resistance, including those against specific antibiotics (light green), RR (dark pink), MDR (dark orange), pre-XDR (red), and XDR (black)

The two Indonesian L3 isolates are confirmed to be clustered with the L3 reference Mtb_NOR1 by the SNPs phylogenomic study (Fig. 3A, yellow arrows). To independently validate the SNPs phylogenomic study, digital spoligotyping was carried out among the multiple MTBC lineages, and the results were consistent with one another (Table 3).

**Table 3:** Spoligotypes of selected control MTBC lineages and Indonesian MTBC L3 strains. Using the Lorikeet pipeline, each forward and reverse sequencing run was digitally spoligotyped [24]

Strain	SRA#	Read#	Spoligotype	Family	Lineage
MtbH37Rv_NED1	SRR23315112	read_1	■■■■■■■■■■ ■■■■■■■■■□ □■■■■■■■■■ ■■□□□□■■■■ ■■■	H37Rv	4
MtbH37Rv_NED1	SRR23315112	read_2	■■■■■■■■■■ ■■■■□□■■■□ □■■■■■■■■■ ■■□□□□■■■■ ■■■	H37Rv	4
Mtb_IDN1	ERR3273086	read_1	■■■■■■■■■■ ■■■■■■■■■■ □□□□■■■■■■ ■■□□□□■■■■ ■■■	LAM9	4
Mtb_IDN1	ERR3273086	read_2	■■■■■■■■■■ ■■■■■■■■■■ □□□□■■■■■■ ■■□□□□■■■■ ■■■	LAM9	4
Mtb_IDN2	SRR12416847	read_1	□□□□□□□□□□ □□□□□□□□□□ □□□□□□□□□□ □□□□■■■■■■ ■■■	Beijing	2
Mtb_IDN2	SRR12416847	read_2	□□□□□□□□□□ □□□□□□□□□□ □□□□□□□□□□ □□□□■■■■■■ ■■■	Beijing	2
Mtb_CHN1	SRR18548950	read_1	□□□□□□□□□□ □□□□□□□□□□ □□□□□□□□□□ □□□□■■■■■■ ■■■	Beijing	2
Mtb_CHN1	SRR18548950	read_2	□□□□□□□□□□ □□□□□□□□□□ □□□□□□□□□□ □□□□■■■■■■ ■■■	Beijing	2
Mtb_IND1	SRR22424726	read_1	□□□□□□□□□□ □□□□□□□□□□ □□□□□□□□□□ □□□□■■■■■■ ■■■	Beijing	2
Mtb_IND1	SRR22424726	read_2	□□□□□□□□□□ □□□□□□□□□□ □□□□□□□□□□ □□□□■■■■■□ ■■□	Beijing	2
Mtb_NOR1	ERR2245380	read_1	■■■□□□□■■□ ■■□■■■■■■□ □□□□□□□□□□ □□□□□■■■■■ ■■■	CAS1-Kili	3
Mtb_NOR1	ERR2245380	read_2	■■■□□□□■■□ ■■□■■■■■■□ □□□□□□□□□□ □□□□□■■■■■ ■■■	CAS1-Kili	3
Mtb_S6970	SRR24682255	read_1	■■■□□□□□□□ □□□□□□□■■■ ■■□□□□□□□□ □□□□■■□□■■ ■■■	CAS1-Delhi	3
Mtb_S6970	SRR24682255	read_2	■■■□□□□□□□ □□□□□□□■■■ ■■□□□□□□□□ □□□□■■□□■■ ■■■	CAS1-Delhi	3
Mtb_S19106	SRR24682254	read_1	■■■□□□□■□■ ■■■■■■■□□□ □□□□□□□□□□ □□□□□□■■■■ ■■■	CAS	3
Mtb_S19106	SRR24682254	read_2	■■■□□□□■□■ ■■■■■■■□□□ □□□□□□□□□□ □□□□□□■■■■ ■■■	CAS	3

### Indonesian MTBC L3 susceptibility to antibiotics

Mtb_S6970 and Mtb_S19106 strains were susceptible to the for first line drugs (Streptomycin, isoniazid, rifampicin and ethambutol) and second line drugs (Ofloxacin, amikacin and kanamycin). Nevertheless, one of the samples, Mtb_S6970, was isolated from a patient who had received a second treatment of rifampicin. The reference L3 isolated in Norway (Mtb_NOR1) was likewise antibiotic-susceptible. Therefore, we evaluated the extended global L3 lineages for their level of antibiotic resistance (Fig. 3B, *n* = 34). In the unrooted phylogenetic tree, the L3 clade is clearly distinguished from the L2 and L4 groups. While the Mtb_S6970 is clustered with three German L3 isolates ERR5979465, ERR5979471, and ERR5979473, the Mtb_S19106 is clearly separated from the Mtb_S6970 to establish its own sub-clade. The Indonesian MTBC L3’s antibiotic susceptibility status, which shows out to be sensitive to all antibiotics, is another crucial finding from the genome analysis. Genome analyses of every collected L3 were checked for potential antibiotic resistance caused by mutations on genes associated with it in order to determine the extent to which such antibiotic sensitivity distributes among MTBC L3. These outcomes were then superimposed on the L3 phylogenetic tree that was created. The tree contrasts L3 strains that are often antibiotic sensitive (shown in blue) with those that are resistant (shown in red). However, the 44% of antibiotic resistant L3 strains are the ones to be concerned about. We further categorize the antibiotic resistance isolates according to whether they are multi- or single-drug resistant (Fig. 3C). The percentages of MDR (from Germany, Iran, Madagascar, UK), Pre-XDR (from China, Germany, Uganda), and XDR (from India) isolates among the resistance isolates were 13.16, 7.89, and 2.63%, respectively (Fig. 3C).

### MTBC L3 virulence gene comparison

**Fig. 4:**
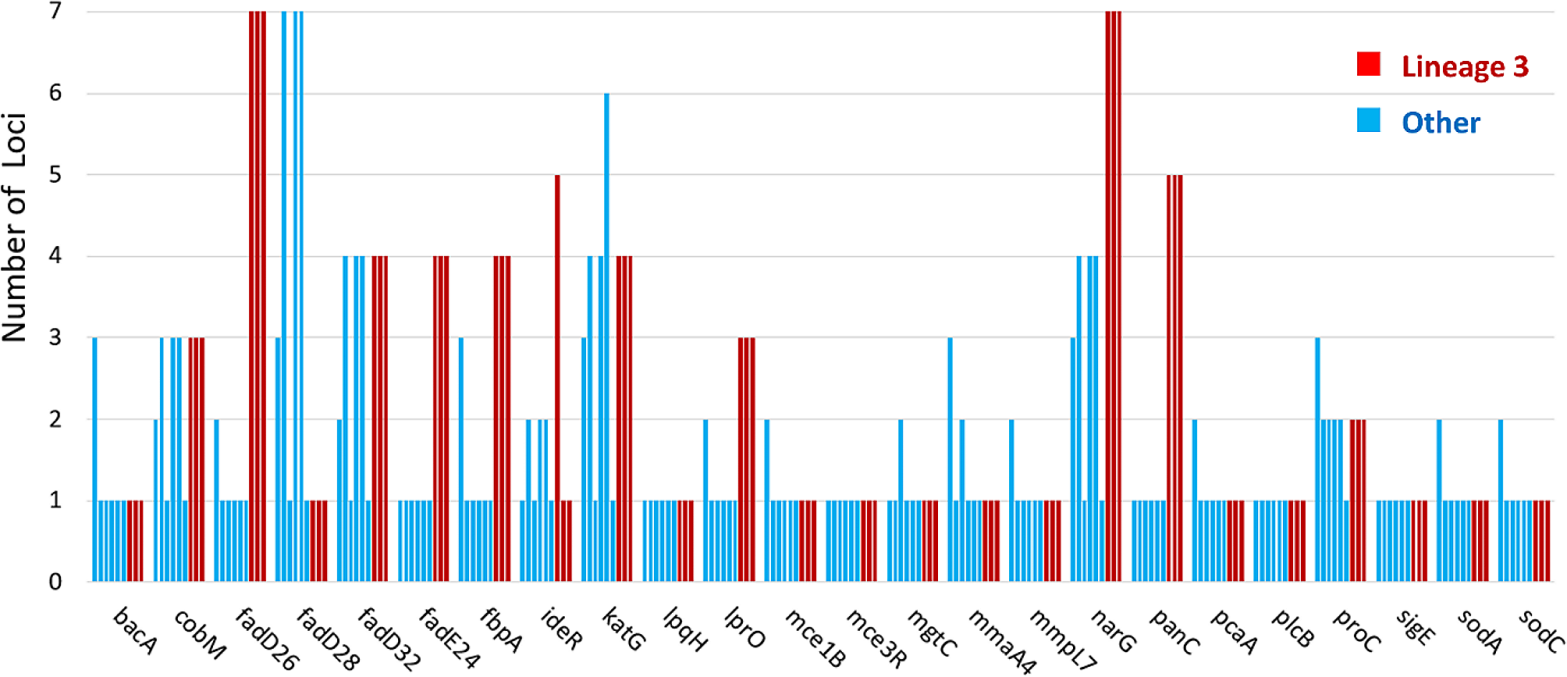
A comparison graph of the examined MTBC genomes’ number of loci for specific virulence genes

According to Smith and Ley’s list of multiple genes implicated in the virulence of *M. tuberculosis*, assembled and annotated genomes of MTBC lineages (Figs. 2A and 3A; Tables 1 and 2) were searched for these genes [9, 10]. The misassembled blocks are colored red, whereas the correct contigs are colored green. The software unicycler was used to assemble the genomes, and circos, a component of the quast genome quality pipelines, was used to create a map of the genomic alignment [11–13]. When long read sequencing employing PacBio or Nanopore technology is utilized to fill gaps between Illumina sequencing reads, the misassembled blocks (in red) would be considerably decreased. Based on the results of the PubMLST genome comparison tool, we discovered that a small number of MTBC virulence genes from L3 strains have a higher number of loci than those from L2s and L4s (Fig. 4). Using the PubMLST’s genome comparator software, it was discovered that 2510 of the 4404 core loci had different numbers of loci [14]. Six of the 24 virulence genes (*lprO, fadE24, fbpA, narG, panC, fadD26*) have more loci in the MTBC L3 lineage (shown in red) than in the other MTBC lineages (shown in blue). The existence of virulence genes, let alone the significant number of loci shared by the two Indonesian MTBC L3 strains, would raise concerns about the infection spreading even though both strains appeared to be antibiotic-sensitive.

## Discussions

Fast molecular DNA tests, such the PCR-based Xpert® MTB/XDR or MTB/RIF, have thus far done an excellent job of compensating for the lengthy wait for results from the gold microbiological standard for diagnosing tuberculosis. Direct evidence of the species of interest at the sequence level has been made possible by the last decades’ rapid development of DNA sequencing technologies, which includes the drop in the cost per unit of sequencing a sample. A correlation between a sequence polymorphism and phenotypes like antibiotic resistance and/or epidemiological grouping may also be possible as a result of various big data analyses. An effective WGS-based MTBC identification of clinical samples from a small area of Mumbai, India, was recently demonstrated in a study that was just published [15]. About 5000 MDR-TB cases are reported in the city of Mumbai each year, which allows the study to account for up to 16% of MDR cases in the area [15]. Briefly said, the research team has amassed a lot of data, including the breakdown of MTBC lineages, antibiotic resistance status, such as MDR, pre-XDR, and XDR, cluster complexes, phylogeographic, and genetic elements involved in the success of L2 MTBC strains in general and clustering strains [15]. Indonesia, with only over 1000 reports, substantially falls short of India’s > 20,000 published reports on tuberculosis. However, the Indonesian government has sequenced more than 2000 isolates from individuals with tuberculosis with the assistance of the World Health Organization, and 1128 of them were screened out for MTBC L3, as reported here. Despite the fact that patient samples were enriched and lab-tested for the right MTBC isolates, all DNA sequences were checked using the kraken2 pipeline to ensure they were categorized in the correct MTBC taxonomic category and free of contamination [16]. The lineages of the sequenced isolates and the level of their antibiotic resistance are determined using the tb-profiler program [17]. A visual representation of the MTBC lineages will be produced via the SNP-based phylogenomics method presented in this study, which makes use of a number of randomly chosen known lineages whose raw WGS data were processed alongside the study samples (Fig. 3A). Separately, the presence or absence of each of the 43 distinct spacer sequences used in conventional wet lab spoligotyping investigations was also determined by digitizing spoligotypes [18]. These digital spoligotypes could then be compared to profiles on SITVITWEB to create a named spoligotype as listed in Table 2 [18]. Both strategies complement one another well. Overall, our WGS data analyses fall within the range of the anticipated *M. tuberculosis* genome profile and characteristics. Whether it be genome size, GC content, predicted coding sequences and their annotated genes, and so on. Importantly for MTBC, mutations associated with antibiotic resistance must be closely examined since they indicate how the population will respond clinically to TB treatment.

Mapping the spread of MTBC lineages across the Indonesian archipelagic region underlined the importance of maintaining a high degree of illness awareness. Here, we showcase the discovery of just two MTBC L3 isolates among over 1280 isolates (to be published elsewhere), which strongly suggests - as it has previously been demonstrated - of the potential global travel-related or even tourism-related transmission. The introduction of L3 to the Indonesian archipelago at its nascent stage of cross-class antibiotic susceptibility just serves to provide a historical perspective on how other prominent lineages in the nation, such as L2 or L4 came to gradually predominate. Unfortunately, the policing of administrative antibiotics that should be used has led to the adaptive phenotypic development of drug resistance, which is exacerbated in the fight against tuberculosis. As history does indeed repeat itself, we are witnessing the spread of future L3 and the ensuing development of drug resistance throughout the archipelagic nation. Only two L3 isolates have so far been identified, both of which are located on islands that are separated from one another by a body of water and are located at a distance of 1400 km (Fig. 1). This fact renders MTBC L3 in Indonesia is still an outlier to the global dominance of L3, which is one of the most commonly distributed lineages in addition to L2 and L4. With further independent incursions into East and North-East Africa, it was hypothesized that the L3 originated in South Asia [19]. Later, L3 distribution in the European Union, Australia, and North America had made a significant indication of the global human migratory movements [19]. The same could be true when we noticed that one of the L3s, the Mtb_S6970 was within the same clonal complexes to three German L3s (Fig. 3B). Given that both the Indonesian and German L3 isolates were sequenced in 2020 and 2021, respectively, predictions concerning a potential global travel-related or even tourism-related transmission may arise from this as well.

Despite the fact that both Indonesian L3s are susceptible to all antibiotics, the characteristics of the host patients are what stand out the most. They were isolated from individuals with tuberculosis who were 55 to 64 years old and of the same gender, but they varied in terms of the host’s medical history in terms of smoking status (Table 2). A patient from Central Java who had been treated and retreated with rifampicin and had been a heavy smoker from the age of 16 was the source of the MTBC L3 strain Mtb_S6970. MTBC L3 strain Mtb_S19106, in contrast, was discovered in a tuberculosis patient from South Sulawesi who had never smoked in his life. The logical question that arises from this phenomenon is whether lineage determination has any bearing on *M. tuberculosis*’s capacity to infect people and cause sickness. Most definitely not. While WGS-based phylogenetic and phylogeographic analyses are crucial for epidemiological purposes and would be essential tools for governmental policing, the medicinal implications of tuberculosis-causing strains depend on the genetic expression of virulence genes. A multi-omics investigation should be part of future research to address the need to understand the underlying genetic expression of virulence genes in patients. Nevertheless, a further query regarding the potential distinction between L3 and L2-L4 in terms of virulence genes level was attempted to be answered in this work. L2 is associated with relapse, fever, and treatment failure as well as a higher resistance than that of L1 or L3. While the majority of MTBC virulence genes typically have the same number of loci throughout lineages, some of those genes (*lprO*, *fadE24*, *fbpA*, *panC*, *fadD26*) in L3 were discovered to be enriched 3 to 7 times (Fig. 4). The lipoprotein gene *lprO* may be implicated in a compensatory strategy for transporting hydrophobic lipid molecules [10]. *FadE24*, a member of the acyl-CoA dehydrogenase subfamily, is intriguing since it may contribute to a new mechanism of drug resistance, albeit this has not yet been determined [10]. One of the mycolyl-transferases, *fbpA*, binds the matrix protein fibronectin as well as transferring long-chain mycolic acids to trehalose derivatives. Interesting macrophage growth patterns that the *fbpA* mutant significantly inhibited provided a target for the creation of a vaccine [9]. *PanC* encodes panthothenate synthetase, whose removal reduces MTBC pathogenicity and would make it a viable target for the development of anti-tuberculosis drugs [9]. Acyl-CoA synthetase, another enzyme involved in fatty acid metabolism, is expressed by fadD26, and its removal reduces the toxicity of MTBC [9]. Consequently, we might infer that the greater number of loci with the potential to overexpress their encoded proteins may make MTBC L3 more virulent.According to L3 can evade the body’s immune response due to its slower growth rate and reduced ability to induce pro-inflammatory factors, thereby maintaining its infectiousness in the population [20]. These could account for why one of the patients who was harboring the MTBC L3 strain Mtb_S6970 did not develop rifampicin resistance during the course of the retreatment.

### Limitations

Several hundred clinical samples. This number is simply too small to comprehend: (i) how did the two L3 isolates develop on two vastly different islands without the two hosts possibly having intimate interactions with one another? (ii) Whether it is possible for L3 to arise from other lineages as a result of a remote incident of evolutionary genetics that is mechanistically unknown. On the other hand, this initial evidence of the L3’s existence corresponds with the epidemiological evolution of other MTBC lineages, which are now firmly established, including L1, L2, and L4, within the population of the archipelagic nation. Therefore, the choice to publish the identification of the two MTBC L3 isolates in this journal takes precedence over years of additional research and fund-raising activities to further screen tens of thousands of clinical samples in order to ascertain the L3’s penetration in Indonesia.

### Electronic supplementary material

Below is the link to the electronic supplementary material.

Supplementary Material 1

## Data Availability

The WGS data generated in this work are available as BioProject PRJNA950554 containing BioSample: SAMN35301034 (Mtb_S6970) and SAMN35082234 (Mtb_S19106) and Sequence Read Archive (SRA) SRR24682255 (Mtb_S6970) and SRR24682254 (Mtb_S19106) in the National Library of Medicine, National Center for Biotechnology Information (www.ncbi.nlm.nih.gov).

## Abbreviations

WGS: whole genome sequencing
MTBC: Mycobacterium tuberculosis complex
EAI: East African-Indian
CAS: Central Asian
MIRU-VNTR: mycobacterial interspersed repetitive units-variable number of tandem repeats
SNP: single nucleotide polymorphism
SNV: single nucleotide variant
