# Supplementary material for: First Indonesian report of WGS-based MTBC L3 discovery
Source: BMC Res Notes. 2024 Jun 25;17:176. doi: 10.1186/s13104-024-06825-5 (PMC11197274; doi:10.1186/s13104-024-06825-5)
Supplement: Supplementary file 1 — Supplementary Material 1 [file 13104_2024_6825_MOESM1_ESM.docx]

**Supplementary**

**Bioinformatic analysis**

Utilizing the public servers at usegalaxy.org or usegalaxy.eu, the sequencing data were uploaded to the Galaxy web platform and analyzed. All raw sequence reads in fastq.gz format was uploaded to the platform. The results of one platform’s analysis were followed up on by analysis on another platform, and so on. On raw sequence data coming from high-throughput sequencing pipelines, a fastqc v.0.11.9 tool was used to perform quality control tests. Then, a program called trimmomatic v.0.38 and coreutils v.8.25 were used to remove low-quality reads and bases from the raw sequencing reads. Prior to performing advanced analyses, it is crucial to run fastqc again to make sure the sequence has reads of an acceptable quality. The next step was to analyze the sequencing reads in order to assign taxonomic labels by comparing the precise k-mer within a query sequence to the least common ancestor of all genomes containing the provided k-mer and searching for contamination DNA using the program kraken2 v.2.1.1.. One of the programs utilized to determine the lineage family of MTBC was digital spoligotyping utilizing the lorikeet v.20 pipeline. A bacterial pipeline called unicycler v.0.5.0 and samtools v.1.15.1 were used to produce a genome assembly utilizing high quality paired sequencing reads from trimmomatic data. Using the quast v.5.2.0, bwa v.0.7.17 and bedtools v.2.30.0 pipelines, the quality of the genome assembly was evaluated. The circos pipeline integrated into the quast package was used to visualize genomic assemblies. The assembled genomes were then annotated using the prokka v.1.14.6 software tool. The jbrowse v.1.16.11 genome browser can then display assembled and annotated genomes produced by unicycler and prokka In order to get ready for a phylogenomic analysis, the program pipeline snippy v.4.6.0 was used to detect SNPs between the reference *M. tuberculosis* ancestral genome and WGS reads, with the minimal fraction for variant evidence reduced from 0.9 to 0.1. To evaluate the quality of aligned reads data against the reference genome, a pipeline qualimap v.2.2.2c was used. A snippy-core tool was then used to merge all SNPs discovered from snippy outputs into a core SNP alignment. A tool within the tb_variant_filter v.0.3.6 pipeline was used to filter out SNPs against the reference MTBC H37Rv template. By region and read alignment depth are the filters that are used. Following the collection of SNPs, tb-profiler v.4.4.1 was used to analyze them in order to find high-quality SNPs known to be associated with antibiotic resistance and identify the lineages of the MTBC strain. Single nucleotide variations (SNVs) can be looked for and found using the same tool within the tb_variant_filter and tbvcfreport v.0.1.10 pipelines to further prepare the sequence data for phylogenomic analysis Filters to apply within the tool are to accept SNVs only and percentage of alt allele with the minimum value of 90% to accept. Then, in order to create a consensus sequence from the obtained variants, the VCF file of the analyzed sequences was subjected to the bcftools consensus v.1.15.1 pipeline. In order to input a multiple sequence alignment to a phylogeny tool, for instance, the consensus sequences are being constructed. Consensus sequences were concatenated, and then the mafft v.7.508 and fasta3 v.36.3.8 program was used to conduct multiple alignments of those sequences. It is possible to feed mafft output from pipelines like concatenate datasets (coreutils) v.8.25, or snippy-core. SNP distance matrix (snp-dists) v.0.8.2 tool can be used to build a matrix for the number of SNP distances between readings in the interim. Using the iqtree v.2.1.2 tool, a phylogenomic or evolutionary tree was finally constructed from a number of sequences. To allow an accuracy test, 1000 bootstrap replicates were done. During this test, the tree was recreated up to 1000 times, each time producing the exact identical branch and location. A program named Newick Display (newick_utils v.1.6 and imagemagick v.7.0.9_6) and a web application called microreact were both used to visualize the phylogenetic tree. Using the interactive web application Genome Comparator from the PubMLST or public databases for molecular type and microbial genome diversity, genomic sequences from different samples were compared.
